# Modeling the Characteristics of Unhealthy Air Pollution Events: A Copula Approach

**DOI:** 10.3390/ijerph18168751

**Published:** 2021-08-19

**Authors:** Nurulkamal Masseran

**Affiliations:** Department of Mathematical Sciences, Faculty of Science and Technology, Universiti Kebangsaan Malaysia, UKM, Bangi 43600, Selangor, Malaysia; kamalmsn@ukm.edu.my; Tel.: +60-3-8921-3424

**Keywords:** air pollution characteristics, pollution risk assessment, statistical modeling

## Abstract

This study proposes the concept of duration (D) and severity (S) measures, which were derived from unhealthy air pollution events. In parallel with that, the application of a copula model is proposed to evaluate unhealthy air pollution events with respect to their duration and severity characteristics. The bivariate criteria represented by duration and severity indicate their structural dependency, long-tail, and non-identically marginal distributions. A copula approach can provide a good statistical tool to deal with these issues and enable the extraction of valuable information from air pollution data. Based on the copula model, several statistical measurements are proposed for describing the characteristics of unhealthy air pollution events, including the Kendall’s *τ* correlation of the copula, the conditional probability of air pollution severity based on a given duration, the joint OR/AND return period, and the conditional D|S and conditional S|D return periods. A case study based on air pollution data indices was conducted in Klang, Malaysia. The results indicate that a copula approach is beneficial for deriving valuable information for planning and mitigating the risks of unhealthy air pollution events.

## 1. Introduction

Air pollution is an important issue that needs to be addressed worldwide, particularly in urban areas. This issue relates to the condition of the air environment corresponding to unhealthy air pollution events [1,2]. Unhealthy air pollution events can be characterized based on their duration and severity. The duration of an air pollution event is determined based on the period in which the air pollution index (API) values are harmful to human health for consecutive periods [3], while severity measures the magnitude of the air pollution event based on the cumulative effect of an unhealthy API during a particular air pollution event. These measures are related because the severity of an air pollution event always depends on its duration. Separate analyses of the duration and severity of an air pollution event cannot reveal their significant association. To model their relationship, joint modeling is required, in which the marginal distributions of both variables are combined [4,5]. Several different bivariate distributions have been proposed by previous researchers in order to model the joint characteristics of bivariate variables. For example, bivariate normal distribution has been applied in many research areas, particularly for the cases where each considered variable can be easily described by a Gaussian/normal distribution [6,7,8,9]. In a similar vein, bivariate lognormal distribution has been employed to describe a phenomenon that can be represented by a lognormal-distributed marginal [10,11]. The Gumbel logistic model [12,13] and the Gumbel mixed model [14,15] were proposed to describe a phenomenon that can be represented by random variables corresponding to a Gumbel-distributed marginal. A bivariate exponential model [16,17] and a Nagao–Kadoya bivariate exponential model [7] have been proposed to model a phenomenon where their marginal random variables can be represented by an exponential model [18]. Apart from that, there are various forms of bivariate gamma models, such as Royen’s Bivariate Gamma model [19], the Izawa Bigamma model [20], the Moran model [21], Schmeiser and Lal’s Bivariate Gamma model [22], and the Loaciga and Leipnik Bivariate Gamma model [23], have been proposed to model the bivariate random variable with a combination of marginal Gamma distribution [24,25,26]. However, if the marginal distribution among the variables is non-identically distributed, obtaining the analytical or closed form of the joint probability model is difficult [27], which makes it challenging to use the available bivariate models to describe the statistical properties of the data. Fortunately, this issue can be resolved using the copula approach [28,29].

Copula functions have the advantage of providing a platform for representing a multivariate distribution for any type or form of marginal variable distribution involved [30,31]. Several researchers have employed the copula model to analyze the air pollution data. Sak et al. [32], for instance, used a copula model to quantify the pollution risk of PM_2.5_ concentration in several cities in China. Chan and So [33] used a copula approach to model extreme spatial air pollution events in Guangdong province, China. Falk et al. [34] used a copula approach to evaluate the joint exceedance probabilities for air pollutants in Milan, Italy. Kim et al. [35] characterized the dependence between several cities in China based on a measure of directional dependence estimated from the PM_2.5_ pollutant variable. Masseran and Hussain [36] modelled and visualized the dependency fluctuation among the air pollution variables in Malaysia using a dynamic Copula approach. He at al. [37] employed a copula model to investigate the time-varying correlations between meteorological factors and atmospheric pollutants in the cities of Beijing and Guangzhou, China. However, most of the available literature focuses on the copula analysis of real values or the magnitude of air pollution events. Thus, this study attempts look at a different perspective by attempting to investigate another important aspect of air pollution events, which is characterized by their duration and severity size. In summary, all of the previous studies found that the copula approach could provide good modeling flexibility for the multivariate distribution of random variables. However, when the dimensionality of the dataset increases, a single copula model parameter is unreliable to describe a simultaneous relationship among the variables [28,29]. To overcome this problem, a vine-copula approach needs to be adopted [38,39]. Fortunately, for the case of bivariate data analysis, the problem of dimensional complexity does not occur [40,41].

A bivariate copula of air pollution severity and duration can effectively reveal the significant relationship between these characteristics. In addition, the copula model can be used to measure the conditional probability and return period of air pollution episodes, which are essential for predicting air pollution events and mitigating their impacts. This information can be beneficial to environmental management authorities in planning for and measuring the risks associated with unhealthy air pollution events. The rest of this paper is organized as follows: Section 2 describes the study area and data. Section 3 provides a description of copula modeling on air pollution characteristics. A variety of copula models are presented in Section 4. In Section 5, an application of flexible parameter estimation and model selection are proposed based on pseudo maximum likelihood approach. In Section 6, the performance of each fitted copula model on the characteristics of unhealthy air pollution data are evaluated. Apart from that, Section 6 also presents several valuable statistical measures for assessing air pollution risk, including (i) the Kendall’s *τ* correlation of the copula to measure the dependency between the duration and severity of an air pollution event, (ii) the conditional probability of a certain air pollution severity given the air pollution duration, (iii) the joint OR/AND return period, and (iv) the conditional D|S and S|D return periods. Finally, in Section 7, the conclusions about the overall findings are provided.

## 2. Study Area and Data

**Figure 1:**
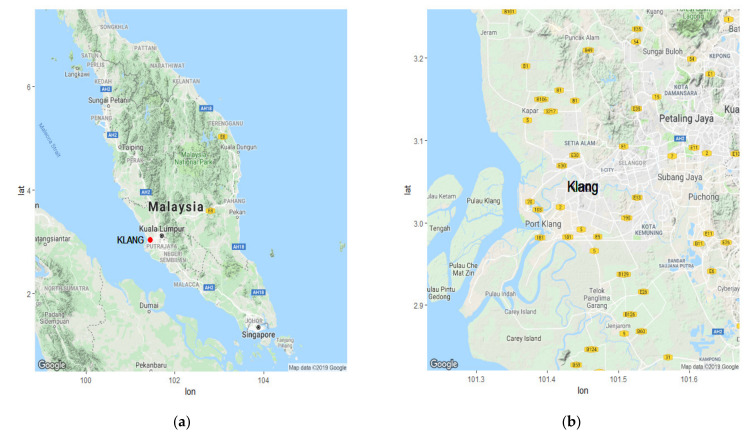
Maps of (**a**) Peninsular Malaysia (Klang location identified by red dot) and (**b**) the city of Klang.

This study investigates the API data in the area of Klang, Peninsular Malaysia, which is located at latitude 101°26′44.023 E and longitude 3°2′41.701 N. Klang is one of the largest cities in Malaysia, with a land area of approximately 573 km^2^. This city has a dense population and is very actively involved in a range of important economic and industrial activities, particularly with the import and export trade. Klang has also been recognized as the 13th busiest trans-shipment port and the 16th busiest container port in the world [42]. Although many advantages are associated with its rapid development, economic activity, and dense population, Klang is prone to poor air quality, which makes it an ideal case for the investigation and continuous evaluation of the Malaysia’s air quality [43]. Figure 1 shows maps of Klang and Peninsular Malaysia [44].

**Figure 2:**
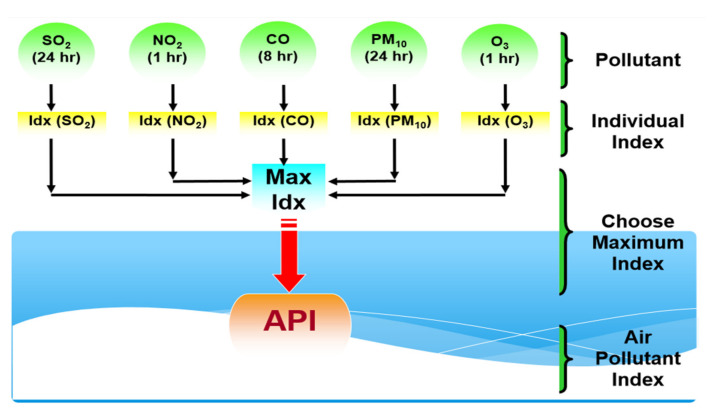
The process of determining the API value.

In Malaysia, the responsibility for collecting, supervising, and reporting API data is held by the Department of Environment (DOE), Malaysia. As a case study, hourly API data in Klang for the period of 1 January 1997 to 31 August 2020 was used in the analysis. A total of five major pollutant variables are recorded hourly, including carbon monoxide (CO), ozone (O_3_), nitrogen dioxide (NO_2_), sulfur dioxide (SO_2_), and particulate matter less than 10 microns in size (PM_10_). The observed data for O_3_, CO, NO_2_, and SO_2_, have been measured in terms of the parts per million (ppm) unit mass of a contaminant, while the observed PM_10_ data have been measured in terms of micrograms per cubic meter (μg/m^3^). Thus, to measure the API indices, these pollutant variables need to be standardized to derive individual indices. Based on each individual indices, their values can then be integrated based on the highest sub-indices to determine the API indices at particular times [45]. A detailed calculation on the standardization and determination of the sub-indices values for each pollutant variable can be referred to in Masseran and Safari [46]. Figure 2 illustrate the schematic process for determining the API value based on five pollutant variables [47].

Based on the API index values, the DOE classifies API values higher than 100 as unhealthy air pollution events [48], and consecutive API values greater than 100 in a given period indicate the duration of an air pollution event. Assuming that the random variable *D_i_* represents the duration of any pollution event, then *i* = 1, 2, 3, …, *n*. Mathematically, the duration of an air pollution event can be determined as follows:

(1) $$D_{i}=\sum_{j=1}^{N}I_{i}\left(API_{j}\right),\;for\;i=1,2,3,\ldots ,n,$$

where *N* is the total number of observations, and $I_{i}\left(API_{j}\right)$ is an indicator function, which is determined as follows:

(2) $$I_{i}\left(API_{j}\right)=\left\{\begin{array}{c} 1,\;if\;API_{j}>100,\\ 0,\;if\;API_{j}\leq 100. \end{array}\right.$$

**Figure 3:**
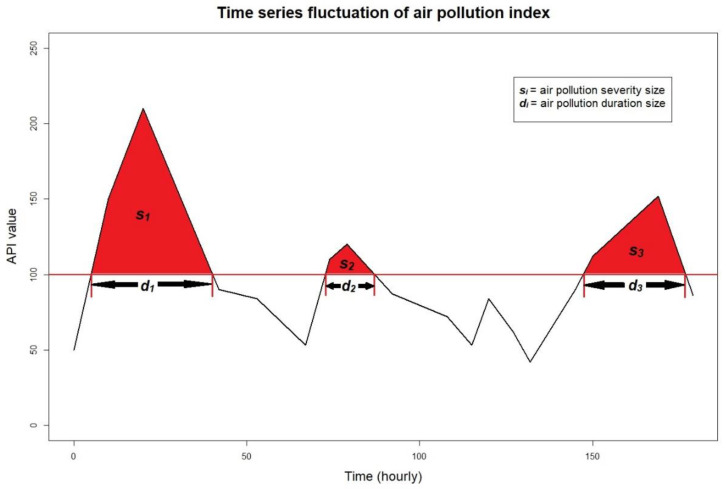
Time series plot corresponding to unhealthy air pollution event threshold.

For any pollution event of a certain duration, the severity of that event can be determined based on the cumulative API values obtained during that period using the following equation:

(3) $$S_{i}=\sum_{j=1}^{D_{i}}API_{j},$$

where *S_i_* is a random variable representing the severity of a pollution event, *i* = 1, 2, 3, …, *n*. Figure 3 shows a graphical representation of the process used to determine the severity and duration of an air pollution event.

## 3. Copula Description of Air Pollution Characteristics

Sklar’s theorem [49] provides an important foundation for the theory and application of the copula model. Copulas play a significant role as mapping functions that enable the combination of uniformly distributed marginal models. Specifically, copulas represent a joint distribution and describe the dependence structure of any arbitrarily distributed dependent variables [50]. Based on Sklar’s theorem, a copula function can be constructed using the following equation:

(4) $$H\left(x_{1},x_{2},\ldots ,x_{2}\right)=C\left[F_{1}\left(x_{1}\right),F_{2}\left(x_{2}\right),\ldots ,F_{n}\left(x_{n}\right)\right]=C\left(u_{1},u_{2},\ldots ,u_{n}\right)$$

where $H\left(x_{1},x_{2},\ldots ,x_{2}\right)$ denotes an *n*-dimensional distribution function, $F_{i}\left(x_{i}\right)=u_{i}$ is a specified univariate marginal distribution function for random variable *X_i_* with $U\sim uniform\left(0,1\right)$, and *C* denotes the copula function [29]. In the context of the bivariate model used in air pollution studies, assuming that the random variables *X_1_ = D* and *X_2_ = S* represent the duration and severity of an air pollution event, respectively, the copula model described in Equation (4) can be simplified as follows:

(5) $$H\left(d,s\right)=C\left[F_{D}\left(d\right),F_{S}\left(s\right)\right]=C\left(u_{1},u_{2}\right).$$

Equation (5) defines a two-dimensional copula as a mapping function of ${\left[0,1\right]}^{2}\to \left[0,1\right]$, which implies that a bivariate distribution function can be defined in ${\left[0,1\right]}^{2}$ with the standard univariate margins $u_{1}$ and $u_{2}$ [31]. Thus, the bivariate density function of a copula can be obtained as follows:

(6) $$c\left(u_{1},u_{2}\right)=\frac{\partial^{2}C\left(u_{1},u_{2}\right)}{\partial u_{1}\partial u_{2}}.$$

Based on the copula model, the dependency between the pollutant duration and severity can be determined using Kendall’s *τ* correlation. This correlation provides a scale-invariant measure for determining this dependency based on the concept of concordance [51]. As mentioned by Klein et al. [52], Kendall’s *τ* has good resistance to outliers and provides a wider class of dependencies. Generally, the Kendall’s *τ* correlation can be related to the copula parameter, *θ*, using the following equation:

(7) $$\tau =4\iint_{\left[0,1\right]}C\left(u,v;\theta \right)c\left(u_{1},u_{2};\theta \right)du_{1}du_{2}-1.$$

Another important piece of information that can be determined is the conditional probability of the air pollution severity given a certain air pollution duration. This information can benefit authorities who are tasked with evaluating the patterns and trends of air pollution behavior, particularly during critical air pollution events. Mathematically, this conditional probability can be computed using the following formula [40]:

(8) $$P\left(S\leq s|D\geq d\prime \right)=\frac{F_{S}\left(s\right)-C\left(F_{D}\left(d\prime \right),F_{S}\left(s\right)\right)}{1-F_{D}\left(d\prime \right)}=\frac{u_{2}-C\left(u_{1}^{\prime },u_{2}\right)}{1-u_{1}^{\prime }}.$$

The copula model can also be used to estimate the return periods of air pollution characteristics. An understanding of the information provided by the return period can serve as a basis for planning and developing monitoring systems to reduce the risk associated with extreme air pollution events. The return period provides insight into the average recurrence interval between unhealthy air pollution events. This information could be beneficial for evaluating the patterns and trends of air pollution behaviors, particularly during critical air pollution events. According to Shiau [40], the copula model is useful for providing information about the joint and conditional return periods of a bivariate event. The joint return period for D≥d or S≥s can be determined as follows:

(9) $$T_{DS}^{\prime }=\frac{E\left(L\right)}{P\left(D\geq d\cup S\geq s\right)}=\frac{E\left(L\right)}{1-F_{DS}\left(d,s\right)}=\frac{E\left(L\right)}{1-C\left(F_{D}\left(d\right),F_{S}\left(s\right)\right)},$$

where $E\left(L\right)$ is the expected inter-arrival time of an air pollution event. Likewise, the joint return period for D≥d and S≥s can be determined as follows:

(10) $$\begin{array}{cc} T_{DS} & =\frac{E\left(L\right)}{P\left(D\geq d\cap S\geq s\right)}=\frac{E\left(L\right)}{1-F_{D}\left(d\right)-F_{S}\left(s\right)+F_{DS}\left(d,s\right)}\\ & =\frac{E\left(L\right)}{1-F_{D}\left(d\right)-F_{S}\left(s\right)+C\left(F_{D}\left(d\right),F_{S}\left(s\right)\right)}. \end{array}$$

On the other hand, the conditional return period for *D*, given S≥s, can be determined as follows:

(11) $$\begin{array}{cc} T_{D|S\geq s} & =\frac{E\left(L\right)}{1-F_{S}\left(s\right)}\times \frac{1}{1-F_{D}\left(d\right)-F_{S}\left(s\right)+F_{DS}\left(d,s\right)}\\ & =\frac{1}{\left[1-F_{S}\left(s\right)\right]\left[1-F_{D}\left(d\right)-F_{S}\left(s\right)+C\left(F_{D}\left(d\right),F_{S}\left(s\right)\right)\right]}, \end{array}$$

Additionally, the conditional return period for *S* given D≥d can be determined as follows:

(12) $$\begin{array}{cc} T_{S|D\geq d} & =\frac{E\left(L\right)}{1-F_{D}\left(d\right)}\times \frac{1}{1-F_{D}\left(d\right)-F_{S}\left(s\right)+F_{DS}\left(d,s\right)}\\ & =\frac{1}{\left[1-F_{D}\left(d\right)\right]\left[1-F_{D}\left(d\right)-F_{S}\left(s\right)+C\left(F_{D}\left(d\right),F_{S}\left(s\right)\right)\right]}. \end{array}$$

## 4. Copula Models

Various copula models are available in the literature for modeling the relationship between the duration–severity variables. However, to obtain an accurate result, comparing several copula models is important to find the one that provides the best representation of the dataset.

### 4.1. Clayton Copula

The Clayton copula model is useful for capturing the positive dependence of bivariate variables in which the strength of the dependency is dictated by the parameter 0≤θ<∞. With a particular rotation, this copula is appropriate for modeling either a positive or negative dependence structure that exists in a dataset [53]. The bivariate joint cumulative distribution function (CDF) and probability density function (PDF) of a Clayton copula are given as:

(13) $$C\left(u_{1},u_{2}\right)={\left(u_{1}^{-\theta }+u_{2}^{-\theta }-1\right)}^{-\frac{1}{\theta }},$$

(14) $$c\left(u_{1},u_{2}\right)=\left(\theta +1\right){\left(u_{1}^{-\theta }+u_{2}^{-\theta }-1\right)}^{-\left(\frac{1+2\theta }{\theta }\right)}{\left(u_{1}u_{2}\right)}^{-\theta -1}.$$

The Kendall’s *τ* correlation determined by the Clayton copula is given as:

(15) $$\tau =\frac{\theta }{\theta +2}$$

### 4.2. Ali–Mikhail–Haq (AMH) Copula

The AMH copula is suitable for datasets in which there is a weaker dependence among the variables [53]. However, this copula is always used for comparison with other copulas in terms of model fitting. The bivariate joint CDF and PDF of the AMH copula are given as:

(16) $$C\left(u_{1},u_{2}\right)=\frac{u_{1}u_{2}}{1-\theta \left(1-u_{1}\right)\left(1-u_{2}\right)},$$

(17) $$c\left(u_{1},u_{2}\right)=\frac{\left[1-\theta \left(1-u_{1}\right)\left(1-u_{2}\right)\right]+2\theta u_{1}u_{2}}{{\left[1-\theta \left(1-u_{1}\right)\left(1-u_{2}\right)\right]}^{3}},$$

With the parameter space [−1,1), the Kendall’s *τ* correlation determined by the AMH copula is given as:

(18) $$\tau =\left[\frac{3\theta -2}{3\theta }\right]-\left[\frac{2{\left(1-\theta \right)}^{2}}{3\theta^{2}}\right]\left[\ln\left(1-\theta \right)\right].$$

### 4.3. Frank Copula

The Frank copula provides a versatile dependency measure because it can accommodate the entire range of dependencies, $\tau \theta \in \left[1,-1\right]$ [41]. Thus, with the correct rotation, the Frank copula is an appropriate model for describing either a positive or negative dependence structure in the dataset [54]. The bivariate joint CDF and PDF of the Frank copula are given as:

(19) $$C\left(u_{1},u_{2}\right)=-\frac{1}{\theta }\ln\left[1+\frac{\left(e^{-\theta u_{1}}-1\right)\left(e^{-\theta u_{2}}-1\right)}{e^{-\theta }-1}\right],$$

(20) $$c\left(u_{1},u_{2}\right)=\frac{-\theta e^{-\theta \left(u_{1}+u_{2}\right)}\left(e^{-\theta }-1\right)}{{\left[e^{-\theta \left(u_{1}+u_{2}\right)}-e^{-\theta u_{1}}-e^{-\theta u_{2}}+e^{-\theta }\right]}^{2}},$$

The Kendall’s *τ* correlation determined by the Frank copula is given as:

(21) $$\tau =1+\frac{4\left[D_{1}\left(\theta \right)-1\right]}{\theta },$$

where $D_{1}\left(\theta \right)=\frac{\int_{0}^{\theta }\frac{t^{k}}{e^{\left(t-1\right)}}dt}{\theta }$ is a Debye function [50].

### 4.4. Plackett Copula

The Plackett copula is also appropriate for modeling either the positive or negative dependence structures in a dataset [53]. The bivariate joint CDF and PDF of the Plackett copula are given as:

(22) $$C\left(u_{1},u_{2}\right)=\frac{\left\{1+\left(\theta -1\right)\left(u_{1}+u_{2}\right)-{\left[{\left(1+\left(\theta -1\right)\left(u_{1}+u_{2}\right)\right)}^{2}-4\theta \left(\theta -1\right)\left(u_{1}u_{2}\right)\right]}^{\frac{1}{2}}\right\}}{2\left(\theta -1\right)},$$

(23) $$c\left(u_{1},u_{2}\right)={\left[{\left(1+\left(\theta -1\right)\left(u_{1}+u_{2}\right)\right)}^{2}-4\theta \left(\theta -1\right)\left(u_{1}u_{2}\right)\right]}^{-\frac{3}{2}}\theta \left[1+\left(\theta -1\right)\left(u_{1}+u_{2}-2u_{1}u_{2}\right)\right]$$

with the parameter space $\left(0,\infty \right)$. However, the Plackett copula has no analytical form for Kendall’s *τ* correlation. Thus, the measure of Kendall’s *τ* correlation must be obtained by the numerical integration of Equation (7) [50].

### 4.5. Gumbel–Hougaard (GH) Copula

The GH copula is suitable to be used to model the positive dependence structure among variables [41,53]. The GH copula can be extended to model the negative dependence by using the concept of rotations [38]. The bivariate joint CDF and PDF of the GH copula are given as:

(24) $$C\left(u_{1},u_{2}\right)=e^{-{\left[{\left(-\ln u_{1}\right)}^{\theta }+{\left(-\ln u_{1}\right)}^{\theta }\right]}^{\frac{1}{\theta }}},$$

(25) $$c\left(u_{1},u_{2}\right)=\frac{C\left(u_{1},u_{2}\right){\left[\left(-\ln u_{1}\right)+\left(-\ln u_{2}\right)\right]}^{\theta -1}{\left[\vartheta \right]}^{\frac{2}{\theta }-2}\left\{\left[\left(\theta -1\right)\right]{\left[\vartheta \right]}^{-\frac{1}{\theta }}+1\right\}}{u_{1}u_{2}},$$

with the parameter space $\left[1,\infty \right)$, and $\vartheta ={\left(-\ln u_{1}\right)}^{\theta }+{\left(-\ln u_{2}\right)}^{\theta }$. The Kendall’s *τ* correlation determined by the GH copula is given as:

(26) $$\tau =1-\frac{1}{\theta }.$$

### 4.6. Joe Copula

The Joe copula is also suitable for describing the positive dependence among variables [41,53]. As reported by McNeil et al. [55], the Joe copula is a good choice for cases in which the dataset exhibits a higher positive correlation among the random variables involved. Apart from that, the Joe copula can be extended to model negative dependence by using the concept of rotations [38]. The bivariate joint CDF and PDF of the Joe copula are given as:

(27) $$C\left(u_{1},u_{2}\right)=1-{\left[{\left(1-u_{1}\right)}^{\theta }+{\left(1-u_{2}\right)}^{\theta }-{\left(1-u_{1}\right)}^{\theta }{\left(1-u_{2}\right)}^{\theta }\right]}^{\frac{1}{\theta }},$$

(28) $$c\left(u_{1},u_{2}\right)=\frac{\partial \left\{1-{\left[{\left(1-u_{1}\right)}^{\theta }+{\left(1-u_{2}\right)}^{\theta }-{\left(1-u_{1}\right)}^{\theta }{\left(1-u_{2}\right)}^{\theta }\right]}^{\frac{1}{\theta }}\right\}}{\partial u_{1}\partial u_{2}},\;$$

with the parameter space $\left[1,\infty \right)$. The Kendall’s *τ* correlation for the Joe copula is obtained as follows:

(29) $$\tau =1+\left(\frac{-2+2\gamma +2\ln\left(2\right)+\Psi \left(\frac{1}{\theta }\right)+\Psi \left(\frac{1}{\theta }\left(\frac{2+\theta }{\theta }\right)\right)+\theta }{\theta -2}\right),$$

where $\gamma =\underset{n\to \infty }{\lim}\left(\sum_{i=1}^{n}\frac{1}{i}-\ln\left(n\right)\right)\approx 0.57721$ is a Euler constant, and $\Psi \left(x\right)=\frac{d}{dx}\ln\left(\Gamma \left(x\right)\right)$ is a digamma function [38].

## 5. Parameter Estimation and Model Selection

Several methods are available in the literature for estimating copula parameters. In this study, the pseudo maximum likelihood estimation (pseudo-MLE) method is used to estimate the parameter of each copula model. This method has the advantage of not requiring accurate information of the parametric form in the marginal model for each variable. Thus, this method provides a more flexible approach than other estimation methods [31].

### 5.1. Pseudo Maximum Likelihood Estimation (Pseudo-MLE)

In general, the pseudo-MLE is a semi-parametric method that uses a nonparametric empirical distribution (NED) to represent the marginal distribution of the variables involved in the copula model [56]. The NED for both the duration and severity variables is determined as follows:

(30) $${\hat{F}}_{i}\left(u\right)=\frac{\sum_{j=1}^{n}1\left(U_{ij}\leq u\right)}{n+1},\;i=1,2.$$

Next, using the NED as a basis for the marginal models, the copula parameter is estimated using the maximum likelihood approach by maximizing the pseudo-log-likelihood function as follows:

(31) $$\log L\left(\theta \right)=\sum_{j=1}^{n}\ln\left[c\left({\hat{F}}_{1}\left(u_{1j}\right),{\hat{F}}_{2}\left(u_{2j}\right);\theta \right)\right].$$

### 5.2. Cross-Validation Copula Information Criterion (cvCIC)

The cvCIC criterion is used to select the copula model that best fits a dataset. Hofert et al. [29] briefly reported that the cvCIC measure for a fitted copula model can be determined as follows:

(32) $$xv_{n}=\frac{\sum_{i=1}^{n}\log\left[c_{\theta_{n,-i}}\left(F_{n,-i}\left(U_{i}\right)\right)\right]}{n},$$

where $\theta_{n,-i}$ represents the maximum pseudo-likelihood estimate, and $F_{n,-i}\left(U_{i}\right)=\left[F_{n,1,-i}\left(u_{1}\right),F_{n,2,-i}\left(u_{2}\right)\right]$ can be computed using the following equation:

(33) $$F_{n,j,-i}\left(u\right)\left\{\begin{array}{c} \frac{\sum_{k=1}^{n}1\left(U_{kj}\leq u\right)}{n},\;\;if\;u\geq \underset{k\in \left[1,2,\ldots ,n\right]\backslash \left[i\right]}{\min}U_{kj},\\ \frac{1}{n},\;\;\;\;otherwise.\;\; \end{array}\right.$$

The copula model that maximizes the cvCIC criterion provides the best-fitted model to the dataset [57]. A detailed discussion of the cvCIC criterion can be found in [58,59].

## 6. Results and Discussion

**Figure 4:**
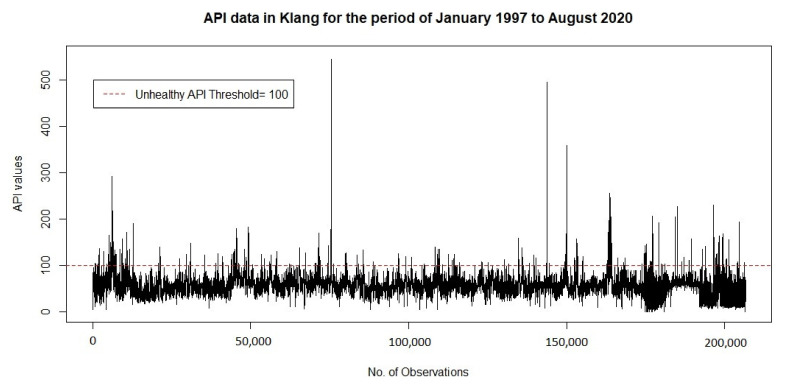
Time series plot corresponds to unhealthy threshold of air pollution events.

Before conducting a detailed analysis of the structure of the dependency of the variables for duration and severity, providing a statistical summary of the dataset is useful. Figure 4 show that the time series plot corresponds to an unhealthy air pollution events threshold in Klang for the period of January 1, 1997 to August 31, 2020. Based on the observed API data, the proportion of unhealthy air pollution events is found to be 2.44%. As described by Masseran and Safari [48], although this proportion is quite small, however, these events provide the most valuable information, particularly on pollution risk management and mitigation.

Table 1 show the descriptive statistics for the severity and duration data. Based on Table 1, the center measures for both variables provided by their means and medians show a large discrepancy, and the measure of spread shows a very large range between the maximum and minimum values. In fact, their standard deviations are also very large, which indicates significant variation in both variables. These findings are more pronounced for severity, but both have significant skewness with a long right-tail distribution. These results are based on measures of skewness and kurtosis. A long right-tail distribution also indicates the presence of extreme and rare events for both variables. 

**Figure 5:**
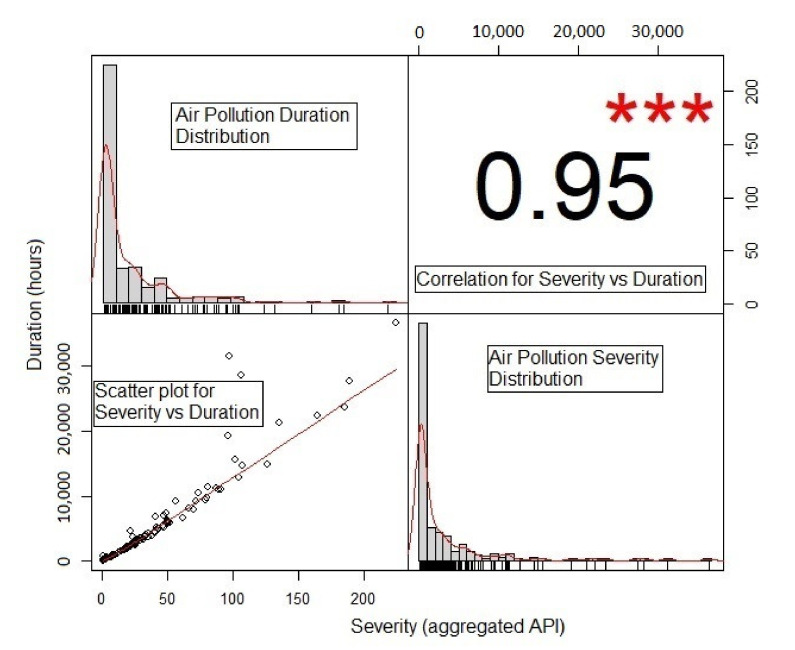
Empirical plots of the relationship of air pollution severity and duration.

**Figure 6:**
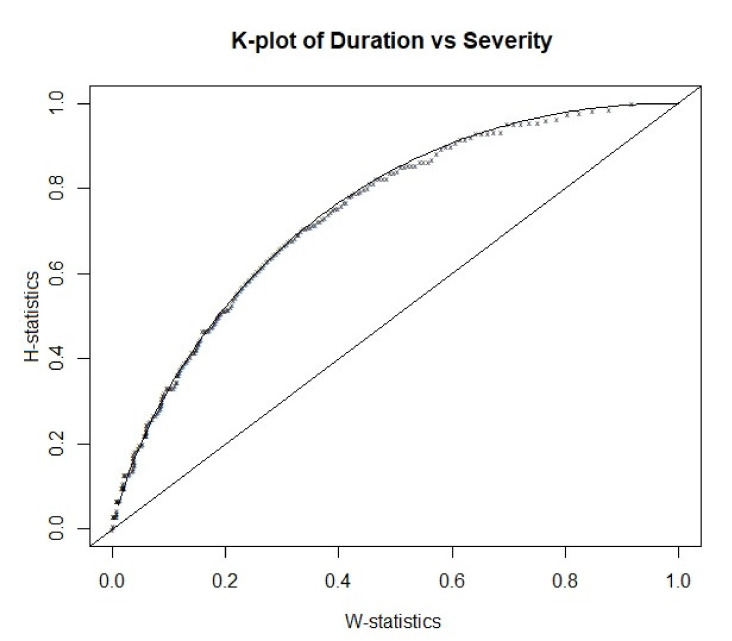
K-plot of the relationship between the duration and severity of air pollution events.

The empirical distribution plots shown in Figure 5 illustrate these findings. The scatter plot in Figure 5 also indicates that the duration and severity have a strong positive dependence, with a Pearson correlation coefficient of 0.95. Note that the asterisks *** indicate the significant correlation at 1% significance level. However, the Pearson correlation measure is not a reliable method for dealing with data that exhibit skewness, long-tail, and non-identically marginal distributions [28]. Kendall’s *τ* correlation, which is derived from a fitted copula model that takes into account the dependence structure among the variables, could provide a more reliable measurement [29]. In addition, some large data points are observed to deviate from the diagonal line, which could be because of the skewness of the data set. To check further, a K-plot is used to reveal the dependent relationships between the points close to the curve, which imply high dependence. If the points are close to the diagonal, this indicates relatively weaker dependence [60]. Figure 6 shows that all of the data points for air pollution severity–duration are very close to the curve, thus confirming the positive relationship between the two.

**Figure 7:**
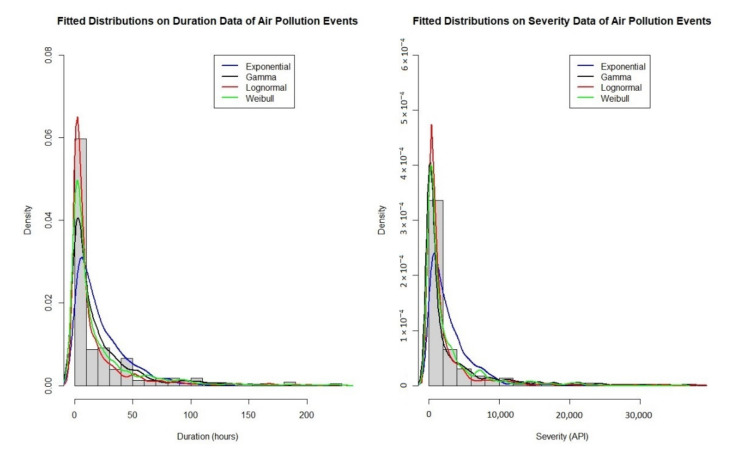
Fitted statistical distributions of duration and severity data.

Based on the empirical statistics and plots, first, the marginal model is determined for the duration–severity data, which serves as a building block for constructing a copula model. As indicated by Table 1 and Figure 5, the distributions of both the duration and severity data are highly skewed to the right. In the literature, the exponential, gamma, lognormal, and Weibull distributions of the models are used to represent marginal models of the duration and severity data [40,61]. Figure 7 shows the fitted marginal distribution used to represent the marginal model. The lognormal and gamma distributions seem to provide an approximate representation of the duration and severity datasets. However, to ensure the accuracy of these fitted models, their goodness of fit needs to be evaluated. 

Table 2 presents the results of the goodness-of-fit evaluation on each of the fitted statistical distributions obtained using the Kolmogorov–Smirnov (KS) test. We found that the *p*-values corresponding to the K–S statistics for all of the fitted distributions of both variables are not significant at α=0.05, which means that the null hypotheses (i.e., the data follow a specified distribution) is rejected. Thus, although the plots in Figure 7 show a good approximation, these fitted distributions cannot reliably represent a marginal model. An accurate marginal model is critical for ensuring precision in copula modeling [62]. To overcome this weakness, an empirical distribution determined using Equation (30) will be used to represent a marginal model. 

**Figure 8:**
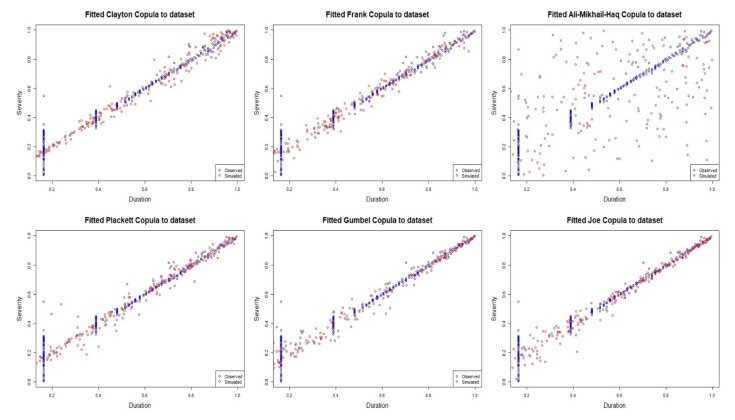
Fitted copula models for the severity–duration dataset.

**Figure 9:**
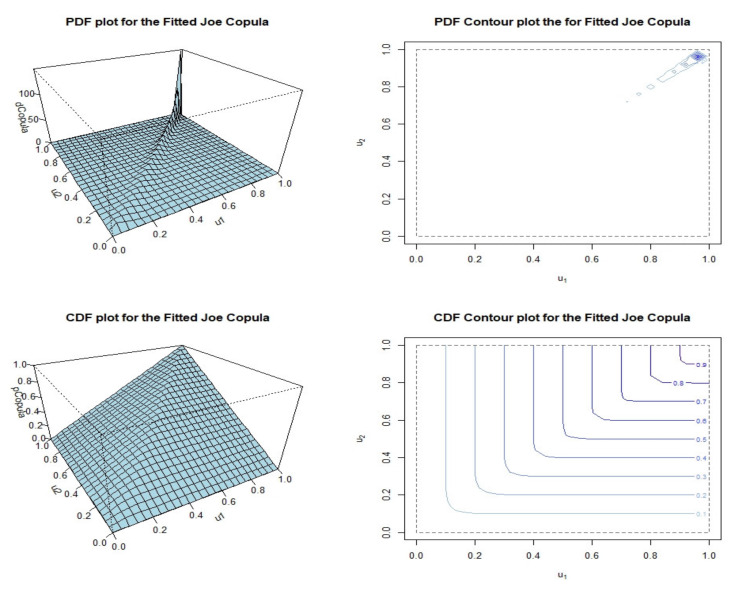
PDF and CDF plots for the fitted Joe copula model corresponding to their contour representations.

Based on the empirical distribution, a pseudo-MLE method is used to estimate the parameters of each fitted copula model for the dataset. Table 3 shows the parameter estimates, cvCIC, and Kendall’s *τ* correlation obtained from each fitted copula model. Based on the estimated parameters for each fitted copula model, the Joe copula model was found to obtain the maximum cvCIC criterion value. Thus, we can conclude that the Joe copula is better than the other copula models in describing the severity–duration relationship. This result is supported by the graphical representation given in Figure 8 of each fitted copula model, in which it is clearly shown that the relationship between the duration and severity of air pollution events is of highly positive dependence with a stronger structural dependency in the right-upper tails of their distributions. Except for the AMH copula model, all of the fitted copula models provide a good representation of the dataset. However, as determined by the cvCIC criterion shown in Table 3, the model that best represents the data is the Joe copula, which has the maximum cvCIC criterion value. The Kendall’s *τ* correlations determined for each copula model do not differ very much, with the exception of that of the AMH copula. The Kendall’s *τ* correlation (0.9195) determined by the Joe copula model is slightly lower that that obtained for the Pearson correlation (0.9500) and is more reliable because it considers the properties of data skewness and long-tail behavior in its computation. Next, based on the fitted copula model, the dependence structure of the severity–duration relationship can be analyzed. This information could benefit the governing authorities who are responsible for managing the risk of extreme air pollution, particularly during critical air pollution events. In addition, the copula approach can work as a simulated model for air pollution risk assessment. For any given air pollution duration size, the analyst can employ the copula model to determine the estimated severity size of air pollution event. Figure 9 shows the PDF and CDF of the fitted Joe copula as density and contour plots. 

**Figure 10:**
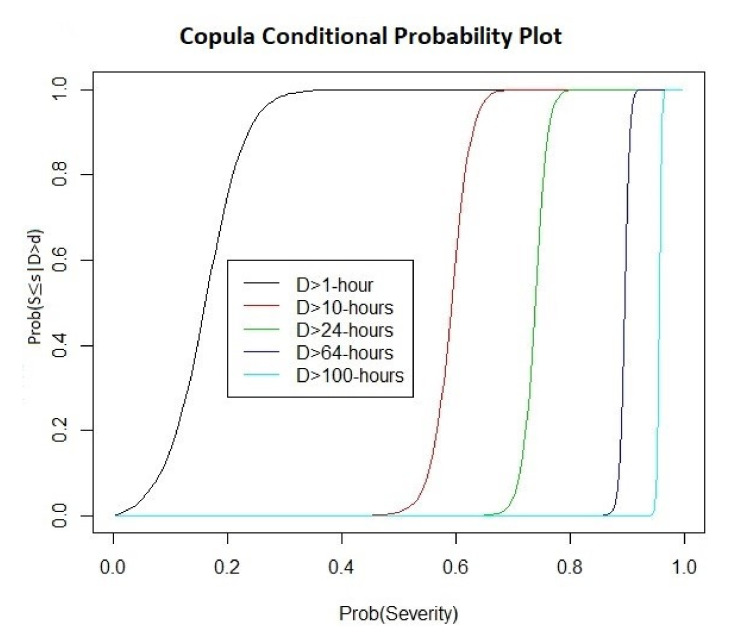
Conditional probability of severity level given the duration.

The PDF and CDF density and contour plots depict the probable behaviors of the duration–severity relationship and confirm the dependence structure shown in the scatter plot of Figure 8. However, the information provided by the PDF and CDF are described with respect to their joint probability distribution of the duration–severity relationship. Therefore, PDF and CDF information are referred to as the likelihood or probability of a certain duration period and severity level occurring simultaneously. The PDF and CDF contour plots efficiently illustrate the positive dependence and stronger structural dependency in the right-upper tails of the duration–severity data. From the fitted Joe copula, the probabilities of air pollution events under certain circumstances with a specific severity or duration can determined. Figure 10 shows the curves of various conditional probabilities of a certain severity level given the duration of an air pollution event. For example, the authorities might want to determine the risk probability for an air pollution severity of less than 8000 and 15,000 for a given duration exceeding 64 h. By normalizing these values using the probability integral transform to obtain a uniform (0,1), the computed probabilities obtained are $\mathrm{Pr}\left(X_{2}\leq 0.90|X_{1}\geq 0.89\right)=0.908$ and $\mathrm{Pr}\left(X_{2}\leq 0.9608|X_{1}\geq 0.89\right)=1$, respectively.

Based on the fitted Joe copula model, the return periods for recurrent air pollution events of different durations and severities can be computed, as shown in Table 4. The rows correspond to the first and second columns in Table 4, indicating given specific levels of duration and severity sizes that represent various scenarios related to air pollution events. On the other hand, all of the values represented in the third, fourth, fifth, and sixth columns indicate the estimated joint and conditional return periods for the air pollution duration–severity pairs in Klang. If the authorities want to evaluate the expected recurrence of air pollution events of a particular duration and severity, four types of information can be determined, as described in Equations (9)–(12). For example, for an air pollution event with a duration of 50 h, with a possibility that this event will achieve a severity level of 20,000, its joint OR return period (D≥50h ∪ S≥20,000) is approximately 34.4 days, and its AND return period (D≥50h ∩ S≥20,000) is approximately 135.4 days. The conditional D|S return period for *D* = 50 h given S≥20,000 is approximately 4153.6 days, and the conditional S|D return period for *S* = 20,000 given D≥50 h is approximately 1056 days. The above information could be very beneficial to the authorities for air pollution even planning and for mitigating the risks associated with unhealthy air pollution events.

## 7. Conclusions

This study proposes the concept of duration and severity measures for evaluating unhealthy air pollution events. Since these bivariate criteria indicate the properties of structural dependency, long-tail, and non- identically marginal distributions, a copula model is proposed to deal with these issues. In particular, this study presents the application of a copula model in evaluating the behaviors of unhealthy air pollution events with respect to their duration and severity characteristics. The duration of an air pollution event was defined as a period in which the air pollution index values qualify as unhealthy for a period of consecutive days. On the other hand, the severity was defined as a magnitude of an air pollution event based on the cumulative effect of an unhealthy API during air pollution events. These two characteristics are related because the severity of an air pollution event always depends on its duration. Thus, considering their dependency, copula models were used to jointly model a combination of the marginal distributions of both variables.

A case study was conducted using data from Klang, Malaysia. To ensure the accuracy of the marginal model, an empirical distribution approach was proposed in the copula model. A total of six types of copula models, namely the Clayton, Ali–Mikhail–Haq, Frank, Plackett, Gumbel–Hougaard, and Joe copulas, were considered for evaluating the relationship between the duration and severity of unhealthy air pollution events in Klang. Based on its efficient fit, the Joe copula was found to best fit the data. Based on the Joe copula model, several valuable statistical measures for assessing air pollution risk were proposed, including (i) the Kendall’s *τ* correlation of the copula to measure the dependency between the duration and severity of an air pollution event, (ii) the conditional probability of a certain air pollution severity given the air pollution duration, (iii) the joint OR/AND return period, and (iv) the conditional D|S and S|D return periods. All of the these measures could be very beneficial for planning and mitigating the risks associated with unhealthy air pollution events. On the other hand, this approach is also applicable in other areas such as for natural hazards, extreme wind speeds, climate change, extreme temperature analysis, etc. Particularly, for the purpose of investigating the severity impact of some particular risky events corresponding to their duration size. Further research is recommended to evaluate how the trivariate relationship corresponds to the duration, severity, and intensity of air pollution event. A vine-copula approach could be used to analyze various combination structures represented by a duration–severity–intensity relationship.

## Figures and Tables

**Table 1:** Descriptive statistics for the severity and duration data.

Variable	Mean	Median	Min. Value	Max. Value	Std. Deviation	Skewness	Kurtosis
Duration (hours)	21.39	3.00	1.00	224.00	64.81	2.78	9.23
Severity	2876.50	367.00	102.00	36,677.00	15,193.68	3.38	13.12

**Table 2:** Goodness-of-fit evaluations of the fitted statistical distributions.

Variable	Fitted Distribution	KS-Statistic	*p*-Value
Duration	Exponential	0.3843	0.0000
	Gamma	0.1834	0.0009
	Lognormal	0.1877	0.0006
	Weibull	0.2227	0.0002
Severity	Exponential	0.3973	0.0000
	Gamma	0.2969	0.0000
	Lognormal	0.1834	0.0009
	Weibull	0.2139	0.0005

**Table 3:** Parameter estimates and cvCIC and Kendall’ *τ* measures obtained by each fitted copula model.

Copula Model	Parameter Estimate (θ)	cVCIC	Kendall’s *τ*
Clayton	22.85	49.108	0.9195
Ali–Mikhail–Haq	1	35.689	0.3333
Frank	48	142.708	0.9195
Placket	846.5	221.530	0.9195
Gumbel	12.42	199.361	0.9194
Joe	25.38	255.258	0.9195

**Table 4:** Joint and conditional return periods for the air pollution duration–severity pairs in Klang using the Joe copula.

Duration Size	API Severity	Joint OR Return Period, $T_{DS}^{\prime }$ (Days)	Joint AND Return Period, $T_{DS}$ (Days)	Conditional D|S Return Period, $T_{D|S\geq s}$ (Days)	Conditional S|D Return Period, $T_{S|D\geq d}$ (Days)
50-h	100	4.4	34.4	34.6	268.5
	1000	10.8	34.4	84.3	268.5
	10,000	34.4	56.4	720.9	439.9
	20,000	34.4	135.4	4153.6	1056.0
	30,000	34.4	406.3	4328.5	3168.0
80-h	100	4.4	56.4	56.7	721.1
	1000	10.8	56.4	138.1	721.1
	10,000	54.8	58.2	743.2	743.2
	20,000	56.4	135.4	4153.6	1730.7
	30,000	56.4	406.3	7382.4	5192.0
100-h	100	4.4	101.6	102.0	2336.4
	1000	10.8	101.6	248.6	2336.4
	10,000	56.4	101.6	1297.9	2336.4
	20,000	101.5	135.5	4153.9	3115.4
	30,000	101.6	406.3	7382.4	9345.6
120-h	100	4.4	169.3	170.0	6490.0
	1000	10.8	169.3	414.2	6490.0
	10,000	56.4	169.3	2163.1	6490.0
	20,000	135.4	169.3	5193.5	6491.8
	30,000	169.3	406.3	7382.4	15,576.0
150-h	100	4.4	225.7	226.7	11,537.9
	1000	10.8	225.7	552.3	11,537.9
	10,000	56.4	225.7	2884.1	11,537.9
	20,000	135.4	225.7	6922.7	11,537.9
	30,000	225.7	406.3	7382.4	20,768.1

## Data Availability

Not applicable.
